# Development and Validation of a Machine Learning System to Identify Reflux Events in Esophageal 24-Hour pH/Impedance Studies

**DOI:** 10.14309/ctg.0000000000000634

**Published:** 2023-08-14

**Authors:** Margaret J. Zhou, Thomas Zikos, Karan Goel, Kabir Goel, Albert Gu, Christopher Re, David Jose Florez Rodriguez, John O. Clarke, Patricia Garcia, Nielsen Fernandez-Becker, Irene Sonu, Afrin Kamal, Sidhartha R. Sinha

**Affiliations:** 1 Division of Gastroenterology and Hepatology, Stanford University, Stanford, California, USA;; 2 Kaiser Foundation Hospitals, Pasadena, California, USA;; 3 Department of Computer Science, Stanford University, Stanford, California, USA;; 4 University of California Berkeley College of Engineering, Berkeley, California, USA.

**Keywords:** 24-hour impedance studies, gastroesophageal reflux disease, machine learning, automated interpretation

## Abstract

**INTRODUCTION:**

Esophageal 24-hour pH/impedance testing is routinely performed to diagnose gastroesophageal reflux disease. Interpretation of these studies is time-intensive for expert physicians and has high inter-reader variability. There are no commercially available machine learning tools to assist with automated identification of reflux events in these studies.

**METHODS:**

A machine learning system to identify reflux events in 24-hour pH/impedance studies was developed, which included an initial signal processing step and a machine learning model. Gold-standard reflux events were defined by a group of expert physicians. Performance metrics were computed to compare the machine learning system, current automated detection software (Reflux Reader v6.1), and an expert physician reader.

**RESULTS:**

The study cohort included 45 patients (20/5/20 patients in the training/validation/test sets, respectively). The mean age was 51 (standard deviation 14.5) years, 47% of patients were male, and 78% of studies were performed off proton-pump inhibitor. Comparing the machine learning system vs current automated software vs expert physician reader, area under the curve was 0.87 (95% confidence interval [CI] 0.85–0.89) vs 0.40 (95% CI 0.37–0.42) vs 0.83 (95% CI 0.81–0.86), respectively; sensitivity was 68.7% vs 61.1% vs 79.4%, respectively; and specificity was 80.8% vs 18.6% vs 87.3%, respectively.

**DISCUSSION:**

We trained and validated a novel machine learning system to successfully identify reflux events in 24-hour pH/impedance studies. Our model performance was superior to that of existing software and comparable to that of a human reader. Machine learning tools could significantly improve automated interpretation of pH/impedance studies.

## INTRODUCTION

Gastroesophageal reflux disease (GERD) is one of the most common conditions seen in gastroenterology practices. The prevalence of GERD is estimated at up to 30% in the United States (1). Current guidelines recommend 24-hour esophageal pH/impedance testing as a method to diagnose GERD (2). Esophageal pH/impedance testing provides information on acid and nonacid reflux events and is one of the most accurate available tests to diagnose GERD. However, interpretation of pH/impedance studies requires expertise and manual review by clinicians, which can be time-consuming. Furthermore, previous studies have demonstrated only moderate concordance between multiple physician reviewers (3–6). Improved tools for augmented or even automated analysis for interpretation of pH/impedance studies may improve both efficiency and diagnostic accuracy.

Currently available automated software for interpreting 24-hour pH/impedance studies has limited diagnostic accuracy. Previous evaluations of available software have reported sensitivity ranging from 73% to 94% for all reflux events with positive predictive values ranging from 62% to 83% (7–9). Furthermore, existing commercial analysis software has not used modern machine learning techniques for development of algorithms or analysis tools to assist with interpretation of these studies. To date, few studies have reported using machine learning methods for interpretation of pH/impedance studies, which include recent studies using a convolutional neural network for study interpretation (10) or decision tree analysis to identify baseline impedance measurements (11).

In this study, we developed and validated a novel machine learning system to identify reflux events in 24-hour pH/impedance studies. We then compared the performance of this system with that of currently available software and expert physician readers.

## METHODS

### Study population

This study cohort included 45 patients who underwent 24-hour pH/impedance testing for evaluation of reflux symptoms at Stanford Health Care from 2019 to 2021 using the VersaFlex LPR ZIND19+8R catheter (Medtronic, Minneapolis, MN). Included patients were either actively on proton-pump inhibitor (PPI) or had discontinued PPI for at least 7 days before the study. A standardized protocol was used in all patients for placement of 24-hour pH/impedance probes. Patients presented to the gastrointestinal laboratory after an overnight fast. A nurse specially trained in the placement of motility catheters first performed an esophageal manometry study to identify the upper border of the lower esophageal sphincter. Once identified, the 24-hour pH/impedance catheter was placed with the distal pH sensor 5 cm proximal to the upper border of the lower esophageal sphincter. For this study, only the LPR ZIND19+8R catheter (Medtronic) was used. This catheter contains a proximal and distal pH sensor, and 2 distal and 4 proximal impedance channels. After placement, patients were given standard instructions for marking meal times, supine position, and symptoms. Patients returned the following day for catheter removal after the passage of 24 hours.

Patients younger than 18 were excluded from the study. The decision was made to perform a 55/45 split of the study cohort to comprise the training/validation and test sets, respectively: 25 patients were included in the training/validation sets (20 patients in the training set and 5 patients in the validation set), and 20 patients were included in the test set. The study protocol for data extraction of pH/impedance studies performed for clinical care was approved by the Stanford University Institutional Review Board (Protocol #51611).

### Development of a machine learning system to identify reflux events

**Figure 1.**
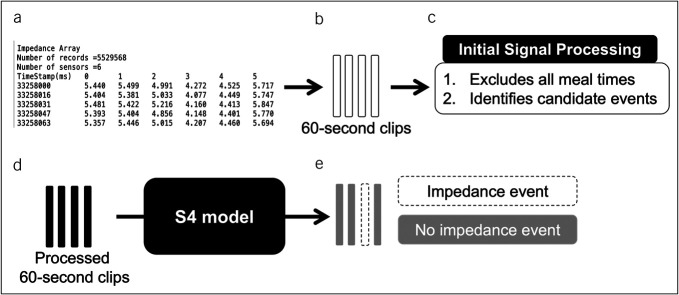
Mechanism of the machine learning system to detect impedance events from 24-hour pH/impedance studies. (**a**) Impedance data are extracted from studies in an array of impedance values at 6 sensors for every timestamp on the order of milliseconds (ms). (**b**) These data are condensed into 60-second clips of impedance data. (**c**) An initial signal processing step excludes all meal times and identifies candidate events by evaluating the impedance clips for decreases in impedance using a peak-finding algorithm. (**d**) The processed clips are inputted into the S4 (stack of structured state space) model. (**e**) The model outputs a prediction for whether an impedance event occurred within each clip.

A diagram of the mechanism of our machine learning system is presented in Figure 1. Raw data from 24-hour pH/impedance studies in the form of a .txt file were first exported from commercially available pH/impedance analysis software (Reflux Reader v6.1; Medtronic). This file contained impedance data organized in an array of impedance values at 6 separate impedance sensors for every timestamp on the order of milliseconds. Impedance values in the data array were then condensed into 60-second clips that underwent an initial signal processing step. This signal processing step excluded all meal times recorded manually by the patient and identified candidate reflux events using a peak finding algorithm that detected decreases in impedance values from more than 1 sensor with high sensitivity. This signal processing step was important in converting the raw impedance data into a format that could be input into our machine learning model.

Using these 60-second clips of impedance data, we developed a machine learning model to classify whether each clip contained an impedance event. The model architecture used was a Structured State Space sequence model (S4). This is a recently developed sequence modeling approach that excels at capturing long-range dependencies within data, making this model well-suited for time series data such as that in pH/impedance studies. The S4 model structure was selected over more commonly encountered deep learning models such as recurrent neural networks or convolutional neural networks because of its unique ability to process lengthy sequential data. Using S4, our machine learning system recognized reflux events based on sharp retrograde changes in impedance measurements from the impedance array. Integrating the available impedance data, the model outputs a probability of whether a reflux event occurred within each 60-second clip.

### Definitions of reflux events and creation of the gold-standard test set

**Figure 2.**
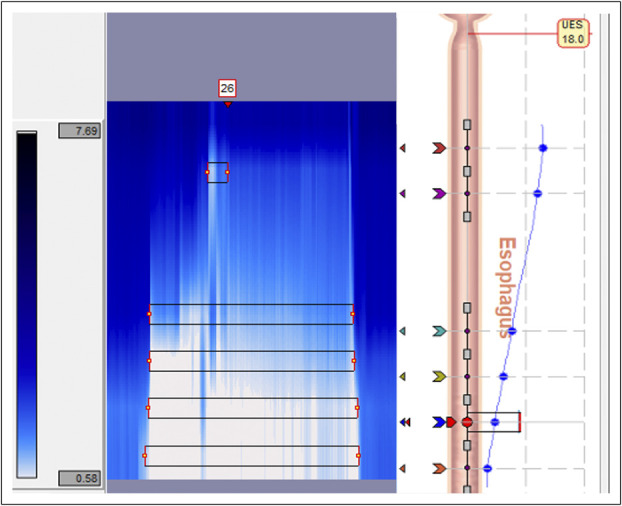
Example of an impedance event in a 24-hour pH/impedance study in Reflux Reader v6.1 (Medtronic). A drop in impedance, signified by the area of white in the impedance diagram, indicates a reflux event. Possible reflux events are identified by the automated software from Reflux Reader v6.1 and labeled numerically. This example shows event number 26. UES, upper esophageal sphincter.

A total of 5 different expert physicians served as readers for the study. Expert readers were physicians at our academic medical center who had specific training in motility disorders and interpretation of motility studies and had at least 1 year of independent practice specializing in motility disorders. For the impedance studies in the training and validation sets, reflux events were identified by the physician reader who originally reviewed the impedance study for standard clinical care. As part of the clinical workflow for pH/impedance study review using Reflux Reader v6.1, uploaded studies underwent initial automated analysis by the Reflux Reader software with identification of candidate events, demarcated by numbered boxes placed above the impedance measurements of a candidate event (Figure 2). Marked candidate events were present in the impedance study at the start of each physician's independent review.

Twenty pH/impedance studies were included in the gold-standard test set, on which performance of our machine learning system was evaluated and compared with performance by the current automated analysis software and an expert physician reader. To create the gold-standard labels for reflux events in the test set, each pH/impedance study was read by at least 2 different physicians. First, the study was evaluated by the physician who originally read the study as part of standard clinical care. Second, a separate physician individually reviewed the entire study (with no knowledge of reflux events identified by the first physician reader). If there was discordance in the identification of a reflux event between the 2 readers, a third separate physician reader was asked to evaluate the discordant events. The assessment made by this third reader of the discordant events was used to define whether the event in question was a reflux event or nonevent in the final gold-standard test set.

Nonevents, or true negative events, in the gold-standard test set were defined as candidate reflux events that had initially been identified by either current automated analysis software or a physician reader as a possible reflux event, but subsequently confirmed to be a nonevent on the gold-standard review. This definition of a nonevent was used to narrow the pool of true negative events in our data set to avoid imbalance between positive and negative labels and to allow for more rigorous evaluation of the specificity of our model.

### Data analysis

For descriptive characteristics, we used unpaired *t* tests and Wilcoxon rank-sum tests to analyze continuous variables. Categorical variables were analyzed using χ^2^ tests or Fisher exact tests for sparse data.

Area under the curve (AUC) was determined by evaluation on the gold-standard test set for our machine learning system, expert physician reader, and current automated analysis software. We used the original physician reader as our human comparator because this review was conducted as part of standard clinical care and thus more likely to reflect real-world physician performance. We also obtained sensitivity and specificity for identification of reflux events by the machine learning system, currently available software, and an expert physician reader regarding performance on the test set. Sensitivity of the machine learning system was calculated by setting a corresponding false positive rate based on the physician reader to obtain a single threshold for sensitivity. Similarly, specificity of the machine learning system was calculated by setting a corresponding true positive rate based on the physician reader to obtain a single threshold for specificity. This strategy for selecting thresholds to compute the sensitivity and specificity of our machine learning system was selected to allow for the most accurate comparison of the performance of our model with that of the expert physician reader. Estimates for the 95% confidence intervals (CIs) for the AUC of the machine learning system were obtained by bootstrapping using 1,000 repetitions of the bootstrap procedure, each of which resampled from the entire test set to produce an estimate of the area under the receiver operating characteristic (12,13).

## RESULTS

### Study population characteristics

Patient characteristics included mean age 51 (standard deviation [SD] 14.5) years, with 47% of patients who were male and 78% of studies performed off PPI. The primary indication for pH/impedance testing was unspecified GERD in both the training/validation (19/25; 76.0%) and test sets (18/20; 90.0%). In the training/validation set, the mean acid exposure time per study was 4.5% (SD 7.9) compared with 3.1% (SD 3.0) in the test set. In the training/validation set, the mean number of reflux events per study was 51.2 (SD 33.0) compared with 45.3 (SD 23.6) in the test set. All baseline characteristics were similar between the training/validation and test sets (Table 1). The Cohen kappa coefficient for interrater reliability between the 2 physician readers was 0.38.

**Table 1.** Baseline patient characteristics for 45 patients undergoing 24-hour pH/impedance testing for reflux symptoms at Stanford Health Care from 2019 to 2021

	Training/Validation set (n = 25)	Test set (n = 20)	*P* value
Age (yr), mean (SD)	51.4 (15.1)	50.4 (14.1)	0.81
Sex (%)			1.00
Male	12 (48.0)	9 (45.0)	
Female	13 (52.0)	11 (55.0)	
Race/Ethnicity			0.11
White	8 (32.0)	11 (55.0)	
Non-White	8 (32.0)	7 (35.0)	
Unknown	9 (36.0)	2 (10.0)	
Study performed on or off PPI (%)			0.12
Off PPI	20 (80.0)	15 (75.0)	
On PPI	5 (20.0)	2 (10.0)	
Primary indication for testing			0.26
GERD	19 (76.0)	18 (90.0)	
GERD postreflux procedure	3 (12.0)	0	
GERD postlung transplant	3 (12.0)	2 (10.0)	
Acid exposure time per study (%), mean (SD)	4.5 (7.9)	3.1 (3.0)	0.47
Impedance events per study, mean (SD)	51.2 (33.0)	45.3 (23.6)	0.52

GERD, gastroesophageal reflux disease; PPI, proton-pump inhibitor; SD, standard deviation.

### Comparison of machine learning system with expert physician readers and current automated analysis software on the test set

**Figure 3.**
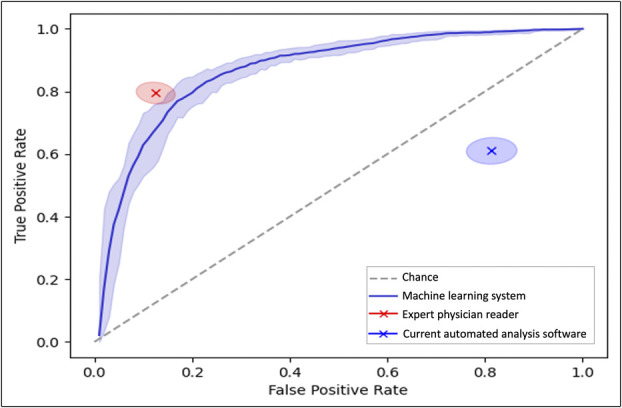
Area under the receiver operating characteristic curves for the prediction of impedance events by the machine learning system. 95% confidence intervals for the area under the receiver operating characteristic values for the machine learning model, expert physician reader, and currently available automated analysis software are signified by the encircled shaded areas corresponding to each point estimate.

The machine learning system demonstrated an AUC of 0.87 (95% CI 0.85–0.89) for the prediction of reflux events in the gold-standard test set. In comparison, currently available automated analysis software demonstrated an AUC of 0.40 (95% CI 0.37–0.42), and the AUC of the expert physician reviewer was 0.83 (95% CI 0.81–0.86) (Table 2, Figure 3).

**Table 2.** AUC, sensitivity, and specificity for identification of reflux events on 24-hour pH/impedance studies by the machine learning system, currently available automated analysis software (Reflux Reader v6.1), and expert physician reader

	AUC (95% CI)	Sensitivity	Specificity
Machine learning system	0.87 (0.85–0.89)	68.7%^a^	80.8%^b^
Current software	0.40 (0.37–0.42)	61.1%	18.6%
Expert physician reader	0.83 (0.81–0.86)	79.4%	87.3%

AUC, area under the curve; CI, confidence interval.

a Based on a false positive rate for the expert physician reader of 13%.

b Based on a true positive rate for the expert physician reader of 79%.

The sensitivity of the machine learning system in identifying reflux events was 68.7% (determined by setting the false positive rate at the level of the expert reader at 12.7%), and the specificity of the machine learning system was 80.8% (determined by setting the true positive rate at the level of the expert reader at 79.4%) (Table 2). In comparison, the sensitivity for the current automated analysis software was 61.1% and specificity was 18.6%. The expert physician reader demonstrated a sensitivity of 79.4% and specificity of 87.3% for identifying reflux events.

## DISCUSSION

In this study, we developed and validated a machine learning system that can successfully identify esophageal reflux events in 24-hour pH/impedance studies with higher AUC compared with currently available automated analysis software and comparable AUC to that of an expert physician reader. Our study describes successful implementation of S4, a novel machine learning model particularly well-suited for lengthy time series data such as pH/impedance data. This deep learning model architecture may be adopted for other medical applications that require sequence modeling.

To the best of our knowledge, this is one of the first studies to use modern deep learning methods for identification of reflux events in 24-hour pH/impedance studies and compare their performance with currently available software and expert physician interpretation. The S4 architecture we used for our machine learning system excels at modeling long sequences and is more computationally efficient than traditional machine learning models that have faced challenges with lengthy sequential data. Furthermore, we used a rigorous definition of gold-standard reflux events and negative events using at least 2 physician consensus to build our test data set on which we compared performance of our machine learning system, current software, and physician readers.

Wong et al recently demonstrated successful use of machine learning methods to identify reflux events in pH/impedance studies (10). They used ResNet-18, a convolutional neural network commonly used for image recognition, to identify reflux events from pH/impedance studies and reported a sensitivity of 84%. Although the sensitivity of their model was superior to that of our machine learning system of 69%, it is difficult to directly compare other metrics between the 2 models as Wong et al did not report area under the receiver operating characteristic or specificity, and furthermore, they did not specify how they defined a true negative event, which could potentially affect these metrics. Nonetheless, their model reports excellent accuracy of 87% and positive predictive value of 86%, which further supports that machine learning techniques can achieve high performance in reflux event identification.

Unlike the models described in our study and by Wong et al that incorporate recent advances in machine learning methods, Rogers et al instead used more traditional decision tree analysis to identify baseline impedance measurements (11). In the process of developing their model, they used their software to identify various events that deviated from baseline to exclude from the data set, including reflux events, swallows, and artifacts. Their model was able to identify 88.5% of these overall events. It is again difficult to directly compare our model's performance with that of Rogers et al because they did not report disaggregated performance solely on reflux events. However, their study provides further support for the use of machine learning techniques in the identification of additional pH/impedance measurements that may benefit from deep learning techniques such as S4.

Previous evaluation of the performance of available automated analysis software has had widely varying metrics and results. Gyawali et al (6) recently demonstrated that in a group of expert physicians interpreting pH/impedance studies (Diversatek, Boulder, CO), the number of reflux events identified by the automated analysis software was significantly higher compared with that of the expert physician readers. This finding is consistent with several previous studies demonstrating overestimation of reflux events by current automated analysis software (5,13). Although the specificity of the current software they used was not reported in their study, their findings suggest that current software likely has insufficient specificity for identifying reflux events with high efficiency. Thus, for our model, we prioritized obtaining high specificity for identifying reflux events, which we achieved with a specificity of 80.8%. Because we aimed to maximize our model's specificity, our model sensitivity was somewhat lower at 68.7%. However, this is comparable with reported sensitivities of available software for identifying reflux events, which has ranged from 73 to 94% for all reflux events and as low as 33% for nonacid reflux events (9,14).

We acknowledge several limitations. Our data set consisted of a retrospectively collected cohort that included some less commonly encountered indications such as postlung transplant GERD, although this was a minority of patients. We did not separately identify acid vs nonacid reflux events, which we plan to develop in subsequent versions of our model. To prevent data imbalance between positive reflux events and true negative events in our test set, we defined negative events as potential reflux events initially identified by either current software or a human reader as a possible event, but later confirmed to be a nonevent on the gold-standard review. This definition of a negative event likely does not reflect real-world use and may represent a more ambiguous set of negative events than may be encountered in everyday clinical practice. Although evaluation on this test set likely underestimates the performance of our model as measured by specificity, this definition of a negative event achieved our goal of obtaining a rigorous estimate of the specificity of our model to minimize false positive events, a frequently encountered challenge with current software. Further iterations of our model to optimize sensitivity will be needed to fit into current physician workflow which typically consists of manual review of events suggested by the software. With the higher specificity of our model, we hope that this pilot study will be a first step in developing a machine learning automated analysis system that will identify impedance events with fewer false positives compared with currently available software. In addition, because we used only the Medtronic software at our institution, we are unable to assess how our model may compare with performance by other commercially available pH/impedance software. Finally, we included a small patient sample size which may limit the generalizability of our results. Larger studies will be needed to validate our findings and provide additional analyses on the use of the machine learning system in specific clinical scenarios, such as among patients who are on vs off PPI. Further studies should also be performed to understand the performance of the machine learning system in high vs low baseline impedance studies, which our study is unable to address because we intentionally excluded studies with very low baseline impedance (less than 750 Ω) for ease of impedance study interpretation in this pilot study.

We have developed a novel machine learning system to identify reflux events in 24-hour pH/impedance studies with superior accuracy compared with current automated analysis software and similar accuracy to an expert physician reader. Furthermore, we used a novel machine learning technique that is well-adapted for time series data such as that seen in impedance studies. This technique can likely be applied to other components required in interpreting pH/impedance studies such as the identification of a swallow and the postreflux swallow-induced peristaltic wave, as well as interpretation of acid vs nonacid reflux events. Using machine learning techniques to improve automated interpretation of pH/impedance studies could have significant benefits for both clinical efficiency and improved diagnostic utility of pH/impedance testing.

## CONFLICTS OF INTEREST

**Guarantor of the article:** Sidhartha R. Sinha, MD.

**Specific author contributions:** M.J.Z.: data collection, data analysis, manuscript preparation; T.Z.: study conception, data collection, data analysis, manuscript preparation; K.G.: data analysis, manuscript preparation; K.G., A.G., C.R., D.R., J.O.C., P.G., N.F.-B., I.S., and A.K.: data collection; S.R.S.: study conception, data collection, data analysis, manuscript preparation.

**Financial support:** None to report.

**Potential competing interests:** None to report.

**Study Highlights**

**WHAT IS KNOWN**

✓ Interpretation of esophageal 24-hour pH/impedance studies is time-intensive.
✓ There are no commercially available machine learning tools to assist with automated identification of reflux events in impedance studies.

**WHAT IS NEW HERE**

✓ We trained and validated a machine learning system to identify reflux events in pH/impedance studies.
✓ Our model's performance was superior to that of existing software and similar to that of a human reader.
✓ Novel machine learning tools may improve automated interpretation of pH/impedance studies.
